# White matter injury detection based on preterm infant cranial ultrasound images

**DOI:** 10.3389/fped.2023.1144952

**Published:** 2023-04-20

**Authors:** Juncheng Zhu, Shifa Yao, Zhao Yao, Jinhua Yu, Zhaoxia Qian, Ping Chen

**Affiliations:** ^1^ School of Information Science and Technology, Fudan University, Shanghai, China; ^2^ Ultrasound Department, The International Peace Maternity and Child Health Hospital, School of Medicine, Shanghai JiaoTong University, Shanghai, China; ^3^ Shanghai Key Laboratory of Embryo Original Diseases, Shanghai, China; ^4^ Radiology Department, The International Peace Maternity and Child Health Hospital, School of Medicine, Shanghai JiaoTong University, Shanghai, China

**Keywords:** white matter injury, cranial ultrasound image, ultrasound radiomics diagnostic system, SDL-Net, MTDL-Net, multi-task learning

## Abstract

**Introduction:**

White matter injury (WMI) is now the major disease that seriously affects the quality of life of preterm infants and causes cerebral palsy of children, which also causes periventricular leuko-malacia (PVL) in severe cases. The study aimed to develop a method based on cranial ultrasound images to evaluate the risk of WMI.

**Methods:**

This study proposed an ultrasound radiomics diagnostic system to predict the WMI risk. A multi-task deep learning model was used to segment white matter and predict the WMI risk simultaneously. In total, 158 preterm infants with 807 cranial ultrasound images were enrolled. WMI occurred in 32preterm infants (20.3%, 32/158).

**Results:**

Ultrasound radiomics diagnostic system implemented a great result with AUC of 0.845 in the testing set. Meanwhile, multi-task deep learning model preformed a promising result both in segmentation of white matter with a Dice coefficient of 0.78 and prediction of WMI risk with AUC of 0.863 in the testing cohort.

**Discussion:**

In this study, we presented a data-driven diagnostic system for white matter injury in preterm infants. The system combined multi-task deep learning and traditional radiomics features to achieve automatic detection of white matter regions on the one hand, and design a fusion strategy of deep learning features and manual radiomics features on the other hand to obtain stable and efficient diagnostic performance.

## 1. Introduction

White matter injury (WMI) is a common brain injury disorder in preterm infants, mostly occurring in preterm infants at 24–35 weeks of gestational age. WMI often leads to various degrees of neurodevelopmental delay, cognitive impairment, and even cerebral palsy in preterm infants. Part of preterm infants with severe WMI only show delayed reaction or visual abnormalities in appearance. WMI in severe cases will cause periventricular leukomalacia (PVL), which is characterized by lesions of deep white matter around the lateral ventricle. PVL is now the major disease that seriously affects the quality of life of preterm infants and causes cerebral palsy of children (1).

Related studies (2) have shown that the incidence of WMI in preterm infants is increasing year by year. It has become one of the most critical diseases in preterm infants, which can lead to long-term neurological sequelae and even death in severe cases. At present, there is no definite and effective treatment plan for this disease, and the clinical goal is mainly focused on early diagnosis and intervention. Imaging is the only diagnostic tool for cerebral white matter injury (3).

At present, imaging is often used to assess the brain injury in preterm infants. Magnetic resonance imaging (MRI), computed tomography (CT), ambulatory eleroencephalography (aEEG), and cranial ultrasound (US) are four main methods for detecting WMI in preterm infants. Among them, MRI is the preferred method. Diffusion weighted imaging (DWI) is a major breakthrough in MRI technology in recent years, which is important in the diagnosis of brain injury in preterm infants (4, 5). However, it is costly, inflexible, and cannot be performed bedside. Some studies have shown that aEEG monitoring is of great value for the early diagnosis of WMI in preterm infants and can effectively assess the status of neurological development in preterm infants (6). Cranial ultrasound has the advantage of being a bedside tool that allows safe, reliable serial imaging. It enables assessment of the evolution of injury over time, as well as brain growth and maturation (7–9).

For PVL in preterm infants, it is recommended that cranial US should be the first choice, and cranial CT should only be used as an adjunctive test due to its radiation properties (9, 10). He Xuehua and Guan Buyun et al. studied quantitative analysis of ultrasonographic gray value in premature infants with PVL (11). They performed threshold segmentation of ultrasound images of PVL in preterm infants, edge extraction of specific region of interest (ROI), quantitative analysis of white matter contained in ROI, and calculation of its average gray value as a quantitative index for evaluation of white matter density in preterm infants. Their study showed that Snake model, a medical image analysis software can provide important information in the early diagnosis and in evaluating the prognosis of PVL. The quantitative analysis of ultrasound gray value is important for the early diagnosis of PVL in preterm infants, which not only improves the accuracy of diagnosis and reduces the operator's subjective judgment error, but also has certain guiding significance for early clinical intervention and reduction of disability.

The diagnosis of WMI by cranial ultrasound imaging is still influenced by interobserver differences. Medical image processing and computer aided diagnosis are being developed to achieve objectivity (9). A semi-automatic ultrasound texture analysis method has been developed to improve the early detection of WMI in newborns (12, 13). The quantitative analysis of ultrasound gray value is important for the early diagnosis of WMI in preterm infants, which not only improves the accuracy of diagnosis and reduces the operator's subjective judgment error, but also has certain guiding significance for early clinical intervention and reduction of disability. But texture analysis method only used little information contained in the cranial ultrasound images.

For ultrasound images, the vast majority of the current clinical practice relies on experienced clinicians' diagnosis. The information of the injury area is extracted according to the intensity and boundary of the echoes. However, different doctors with different clinical experience will have different conclusions from the same cranial US images. Therefore, the standardized evaluation of WMI based on cranial US images has clinical application value. Our study aims to provide a more accurate and comprehensive method based on cranial US images for the early diagnosis and treatment of WMI in preterm infants, in order to reduce the subjectivity of analysis errors caused by human factors. Therefore, we tried to present a diagnostic system for white matter injury in preterm infants. The system combines multi-task deep learning and traditional radiomics features to achieve automatic detection of white matter regions and obtain stable and efficient diagnostic performance of WMI.

## 2. Materials and methods

### 2.1. Patients

The data used in this study was obtained from the International Peace Maternity & Child Health Hospital. Ethical approval was waived because it was a retrospective study. In this study, 158 preterm infants with cranial US images were obtained using Philips ultrasonic instrument (EPIQ5 or CX50) with convex array probe (C8-5; 5–8 MHz). The inclusion criteria were: (1) gestational age < 37 weeks, age ≤ 7 days, (2) preterm infants underwent cranial ultrasound diagnosis and had clear B-mode ultrasound images (B-US), (3) birth mother had obstetric complications, (4) preterm infants had a clear expression of intrauterine distress or a clear history of asphyxia during labor, (5) shortly after birth, preterm infants presented with persistent increased anterior fontanelle tone and neurological symptoms.

**Figure 1:**
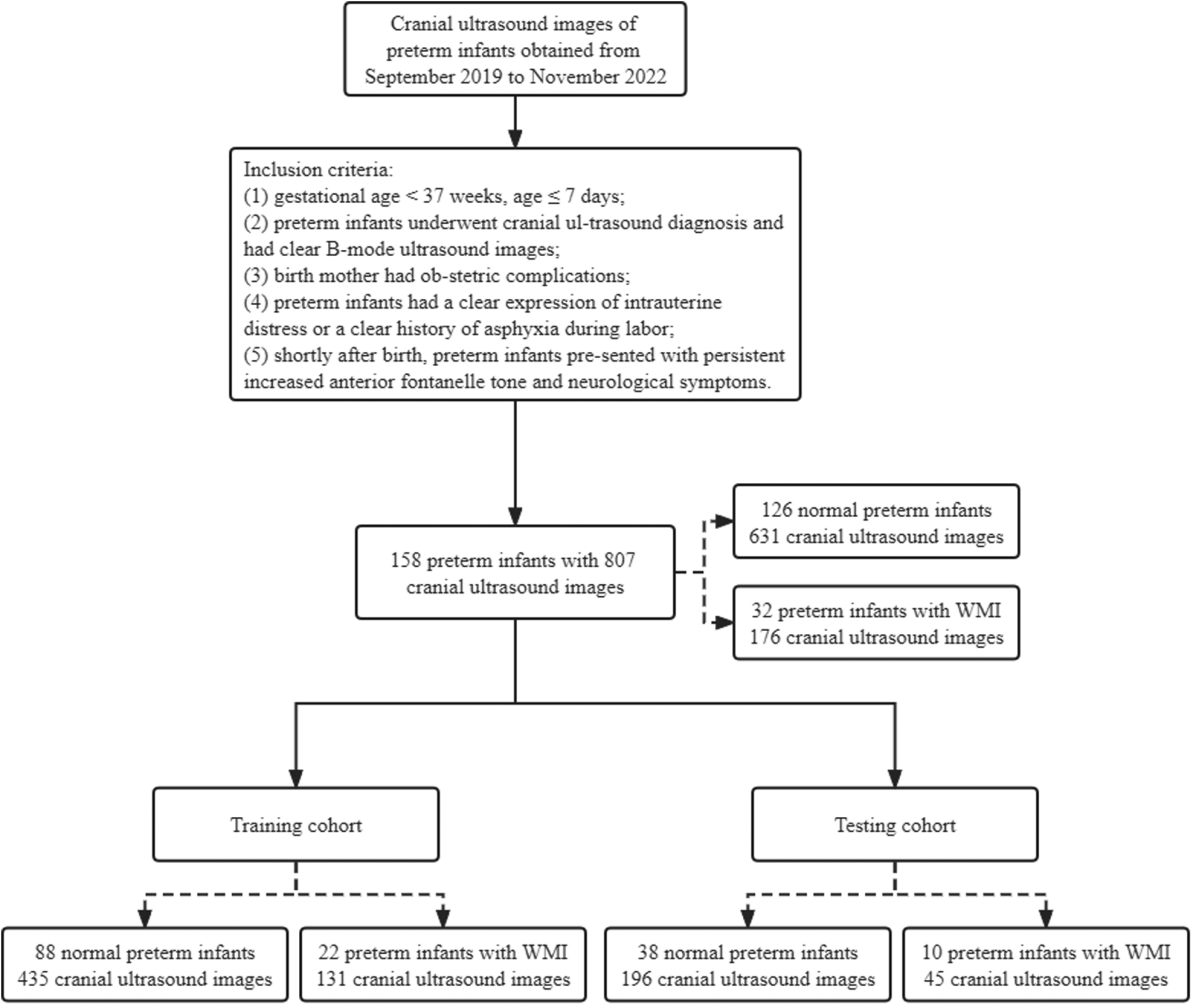
Overview of the patients of the study.

The study population consisted of 158 preterm infants with 807 cranial US images. 126 cases in the data set were normal preterm infants and 32 cases were preterm infants with WMI. We split the data set into training cohort (110 preterm infants with 566 cranial US images) and testing cohort (48 preterm infants with 241 cranial US images) at a ratio of 7:3. Figure 1 presents an overview of the patients of the study. The training cohort consists of 88 normal preterm infants (435 cranial US images) and 22 preterm infants with WMI (131 cranial US images). The testing cohort consists of 38 normal preterm infants (196 cranial US images) and 10 preterm infants with WMI (45 cranial US images).

### 2.2. Cranial ultrasound images

Ultrasonography is a technique that uses specific sound waves to measure the data of morphology and physiology of tissue structures to understand the structure and pathological processes in the human body. B-mode ultrasound (B-US) is a two-dimensional image that is displayed on the screen in grayscale in real-time. With the clinical application of ultrasonic diagnosis technology, the technology began to be used in the diagnosis of neonatal intracranial diseases in the late 1970s. Since the fontanelle is not yet closed at birth, it provides unique diagnostic conditions for clinical ultrasound detection of intracranial lesions (14). The newborn's cranial ultrasound technology is a unique diagnostic tool. The development of neonatal cranial ultrasound technology has opened up a new way for clinicians to understand intracranial lesions *in vivo*. Cranial ultrasound is non-invasive, painless, inexpensive, simple, easy to perform and intuitive, and has become an indispensable routine method for the diagnosis of intracranial diseases in high-risk infants.

The anterior fontanelle of coronal plane set as the standard plane of measurement. This plane clearly shows the bilateral lateral fissure, corpus callosum, and anterior horn of the lateral ventricle. In each preterm infant, 3–6 ultrasound images were obtained in this plane. In total, we obtained 807 ultrasound image samples from 158 preterm infants. All ultrasound examinations were performed by the 3 radiologists with at least 10 years of experience. And all data had the golden standard of manual segmentation by same radiologist.

### 2.3. Ultrasound radiomics diagnostic system

**Figure 2:**
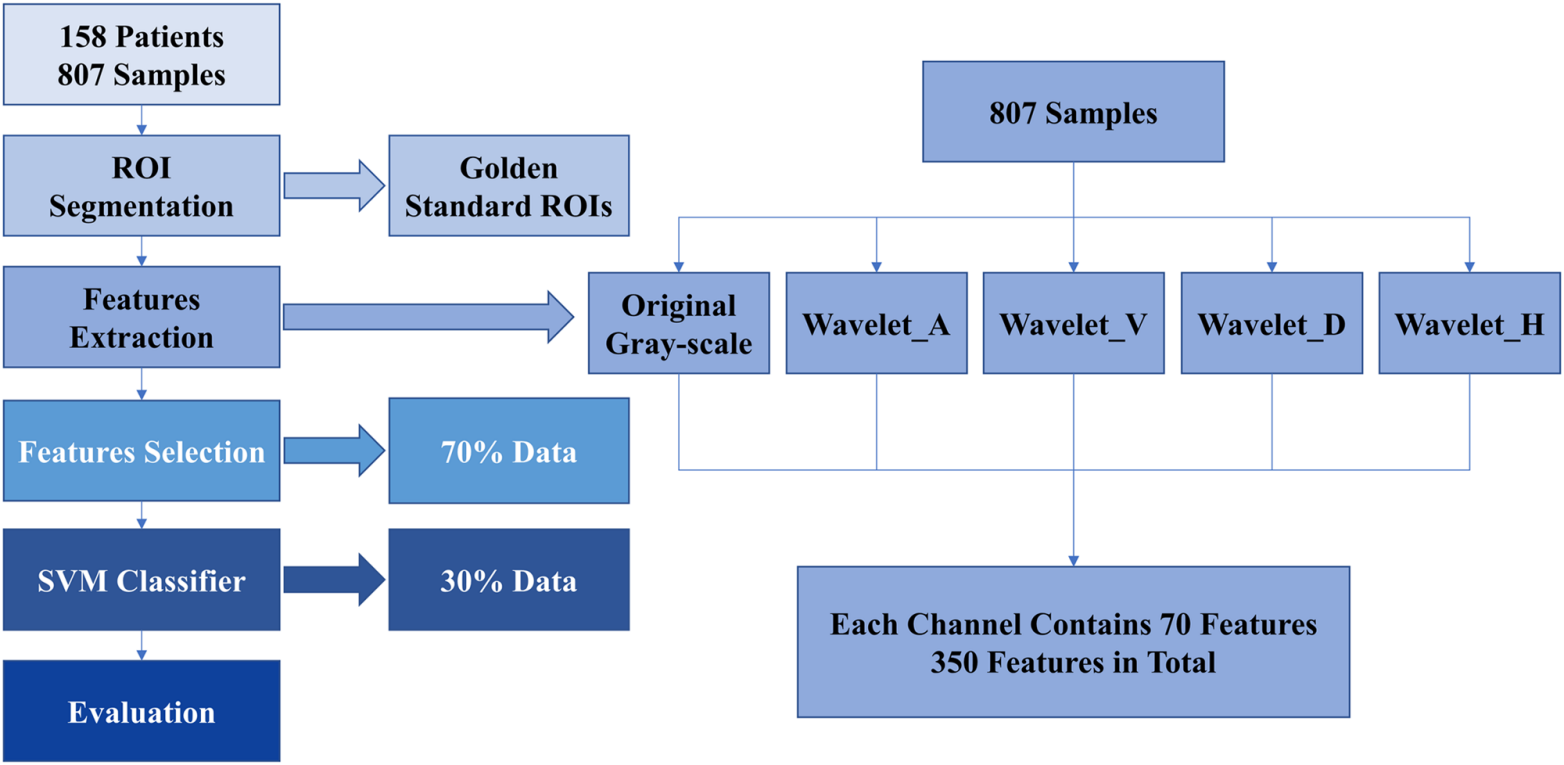
Overview of our ultrasound radiomics diagnostic system.

We used computer-aided diagnostic system based on B-US for the prediction of WMI in preterm infant. There are mainly four steps: ultrasound image segmentation, feature extraction, feature selection, and classification. The overview of our ultrasound radiomics diagnostic system is presented in Figure 2.

We used the golden standard segmented manually by radiologists as the input ROIs. Features were extracted from each ROI. Then, we transferred B-US ROIs to Wavelet_A, Wavelet_V, Wavelet_D, and Wavelet_H modes (15). In each mode, 70 features were extracted from each ROI. Finally, we obtained a total of 350 features in each B-US ROI, including 70 basis features and 280 wavelet features. Among them, the 70 basis features are as follows: 16 histogram features, 23 grey-level co-occurrence matrix (GLCM) based features, 13 gray-level run length matrix (GLRLM) based features, 13 gray-level size zone matrix (GLSZM) based features, and 5 neighboring gray tone difference matrix (NGTDM) based features. The details are shown in Table 1.

**Table 1:** 350 features extracted from B-US.

Feature Type	Feature Name	Number of characteristics
Basic Features	**Total number of base features**	**70**
Histogram	1) Energy 2) Total Energy 3) Entropy 4) Minimum 5) Maximum 6) Mean 7) Median 8) Interquartile Range 9) Standard Deviation 10) Mean Absolute Deviation 11) Range 12) Root Mean Squared 13) Uniformity 14) Variance 15) Kurtosis 16) Skewness	16
GLCM	17) Autocorrelation 18) Joint Average 19) Cluster Prominence 20) Cluster Shade 21) Cluster Tendency 22) Sum Variance 23) Difference Average 24) Correlation 25) Difference Entropy 26) Homogeneity 1 27) Homogeneity 2 28) Sum Average 29) Dissimilarity 30) Joint Energy 31) Informational Measure of Correlation 1 32) Informational Measure of Correlation 2 33) Inverse Difference Moment 34) Joint Entropy 35) Maximal Correlation Coefficient 36) Inverse Difference 37) Inverse Variance 38) Maximum Probability 39) Difference Variance	23
GLRLM	40) Short Run Emphasis 41) Long Run Emphasis 42) Gray Level Non-Uniformity 43) Run Length Non-Uniformity 44) Run Percentage 45) Low Gray Level Run Emphasis 46) High Gray Level Run Emphasis 47) Short Run Low Gray Level Emphasis 48) Short Run High Gray Level Emphasis 49) Long Run Low Gray Level Emphasis 50) Long Run High Gray Level Emphasis 51) Gray-Level Variance 52) Run-Length Variance	13
GLSZM	53) Small Area Emphasis 54) Large Area Emphasis 55) Gray Level Non-Uniformity 56) Size-Zone Non-Uniformity 57) Zone Percentage 58) Gray Level Variance 59) Zone Variance 60) Low Gray Level Zone Emphasis 61) High Gray Level Zone Emphasis 62) Small Area Low Gray Level Emphasis 63) Small Area High Gray Level Emphasis 64) Large Area Low Gray Level Emphasis 65) Large Area High Gray Level Emphasis	13
NGTDM	66) Coarseness 67) Contrast 68) Busyness 69) Complexity 70) Strength	5
Wavelet Characteristics	Wavelet transform was performed on the original B-US image to obtain wavelet_A, wavelet_H, wavelet_V and wavelet_D modes, and then the basic features in the transformed modes were extracted.	280
	**Total number of features extracted from cranial US images**	**350**

We used sparse representation-based classifier (SRC) for feature selection, which was proposed by Li (16). The main idea of SRC is to identify the most relevant features based on the importance index generated in each bootstrap iteration (17). The SRC is based on the assumption that all signals can be represented by a linear combination of atoms in the dictionary. The atoms used to represent the signals are equivalent to the significant features. And the features interact with each other during the representation of the signals. The coefficients of the representation correspond to the importance of the features. All features can be ranked according to their coefficients. So, the top 20% of the atoms with a prominent role are retained as significant features.

In this study, support vector machine (SVM) based cost-support vector classifier (C-SVC) was used to predict the WMI risk by selected features. SVM is a binary classification model and also a linear classifier defined on a certain feature space. By using kernel functions, SVM can be transformed into a nonlinear classifier (17). The purpose of SVM is to split the sample by finding a hyperplane. C-SVC using kernel functions has been widely used in machine learning for high-dimensional complex feature spaces especially when the samples are not balanced. In the process of searching the optimal hyperplane in high-dimensional space, C-SVC has good discriminative power and stability (18).

### 2.4. Deep learning modeling

Deep learning models have been widely used in clinical medicine, and it is witnessing increasing innovations in the fields of AI-aided image analysis, AI-aided lesion determination, AI-assisted healthcare management, and so on (19, 20). In this study, we used two deep learning models to implement segmentation of white matter and prediction of WMI risk of preterm infants.

#### 2.4.1. Segmentation deep learning model

**Figure 3:**
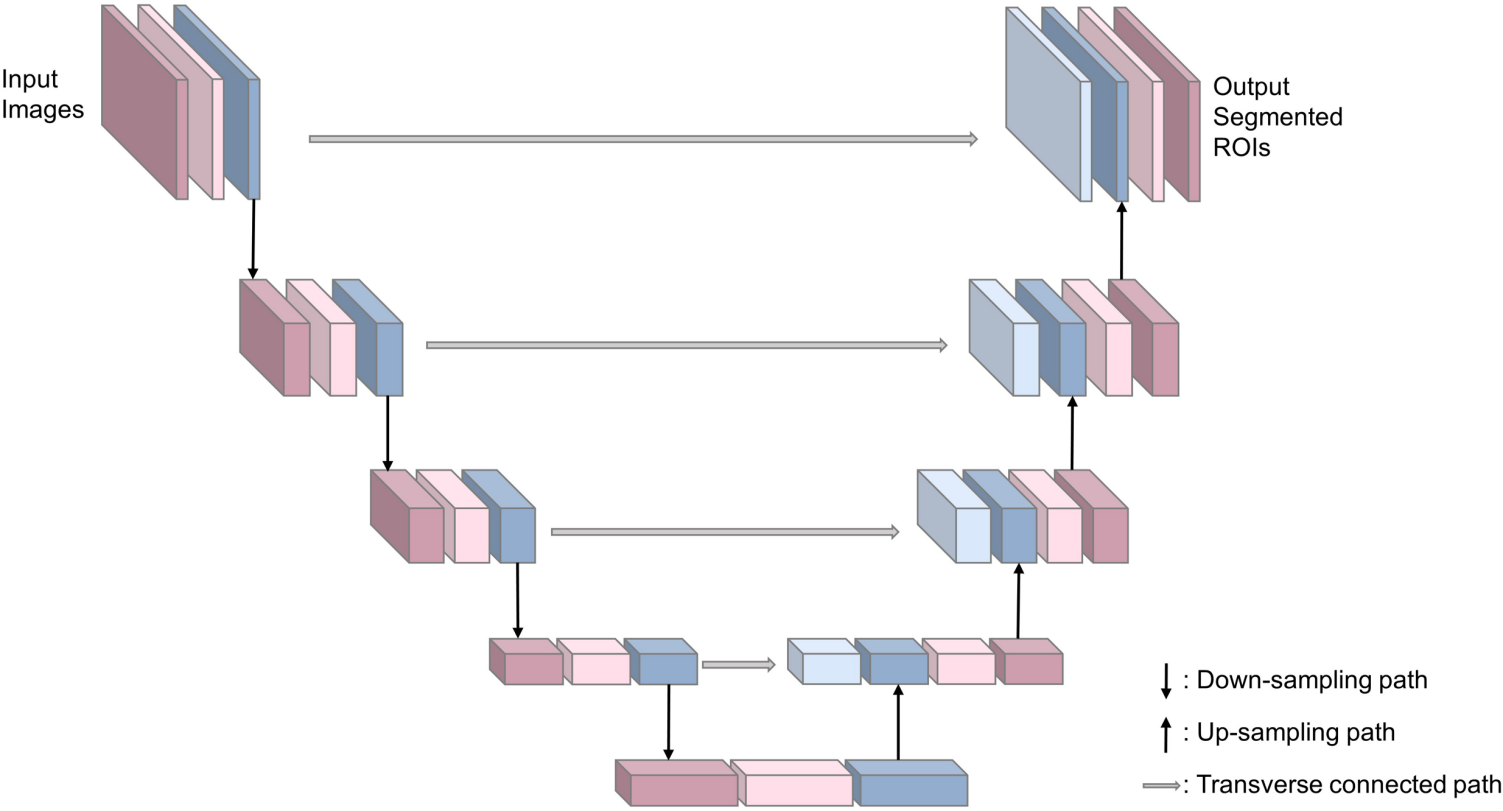
Structure of SDL-Net.

In order to find the most suitable segmentation deep learning network (SDL-Net), we compared FCN (21), U-Net (22), TransUNet (23) and Swin-Unet (24) for white matter segmentations. Based on the result of this ablation experiment (detailed in Supplementary Material), we used U-Net as our SDL-Net. U-Net is an improved segmentation network based on FCN (21) that uses only a small amount of data to train an end-to-end (image in, image out) network and works well for medical images. U-Net network includes a down-sampling path for capturing semantic information, an up-sampling path for accurate segmentation localization, and a transverse connected path. The structure is shown in Figure 3. The down-sampling path is used to extract features of the image at different scales. The up-sampling path is symmetric to the down-sampling path, and they form a U-shaped structure. The transverse connected path allows the network to propagate information of the original image directly to the higher level of high-resolution layer. This structure makes the output layers of the whole network with no missing context information comparing with the original images, and allows U-Net to be used for segmentation of small datasets and reduce the memory limitation of the graphics processing unit (GPU).

#### 2.4.2. Multi-task deep learning model

**Figure 4:**
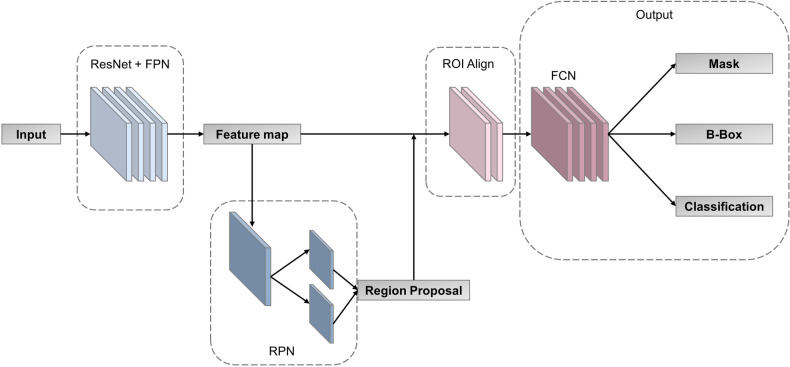
Structure of our multi-task deep learning model.

In order to find the most suitable multi-task deep learning network (MTDL-Net), we compared Fast R-CNN (25), Faster R-CNN (26), Mask R-CNN (27) and RS-Net (28) for white matter segmentations and WMI status prediction. Based on the result of this ablation experiment (detailed in Supplementary Material), we used Mask R-CNN as our MTDL-Net. Mask R-CNN is an improved convolutional neural network proposed based on Faster R-CNN (25). It can accomplish target detection and semantic segmentation simultaneously. The structure of our multi-task deep learning model is divided into three main parts, which is shown in Figure 4. The first part is feature pyramid networks (FPN) (29), which is used for feature map extraction from the whole image. The second part is region proposal network (RPN) (26), which is used for ROI proposal generation. The third part is output layer for classification, bounding box (B-Box) detection, and ROI mask segmentation. There is an ROI Align layer between the second and third parts for obtaining the feature maps corresponding to each ROI. Our multi-task deep learning model has three final outputs: classification labels, B-Box, and semantic segmentation ROIs.

### 2.5. Statistical analysis

The AUC was used as an index for the evaluation of the diagnostic performance. The sensitivity (SENS), specificity (SPEC), accuracy (ACC), and positive and negative diagnostic likelihood ratios (LR+, LR−) were calculated. We adopted Dice coefficient (Dice) and Intersection over Union (IoU) to evaluate the performance of segmentation. The definitions of these indexes are detailed in Supplementary Material.

The statistical analyses were performed using MedCalc software (version 20.027; MedCalc Software bvba, Mariakerke, Belgium), SPSS software (v. 22.0; IBM Corp., Armonk, NY, USA), Python 3.7, and Matlab R2020b.

### 2.6. Implementation details

While training, we implemented an asymmetric cyclic sampling strategy to optimize the unbalanced dataset and augment the training dataset. In order to improve the generalization ability considering the limited medical data, we applied oversampling strategy to augment the data which belong to preterm infants with WMI during training. Additionally, the training set was artificially augmented by random translation, rotation and flipping from the original images to prevent overfitting of the networks due to the limited training dataset. More details about data augmentation techniques are described in Supplementary Material.

In SDL-Net, the max training epoch is set to 100 with a batch size of 4 empirically. The model parameters are updated *via* the Adam optimizer with a learning rate of $1\;*\;e^{-3}$. In MTDL-Net, the max training epoch is set to 100 with a batch size of 4 empirically. The model parameters are updated *via* the Adam optimizer with a learning rate of $2\;*\;e^{-4}$.

Our network was implemented in the PyTorch platform, and MATLAB was used to realize data pre-processing. The entire training process was performed on a computer equipped with an Intel Xeon 4210 CPU with 128 GB RAM, and a Nvidia GeForce RTX 2080 Ti.

## 3. Results

### 3.1. WMI prediction based on ultrasound radiomics diagnostic system

In this part, the golden standard ROIs segmented by radiologists were used in feature extraction.

**Figure 5:**
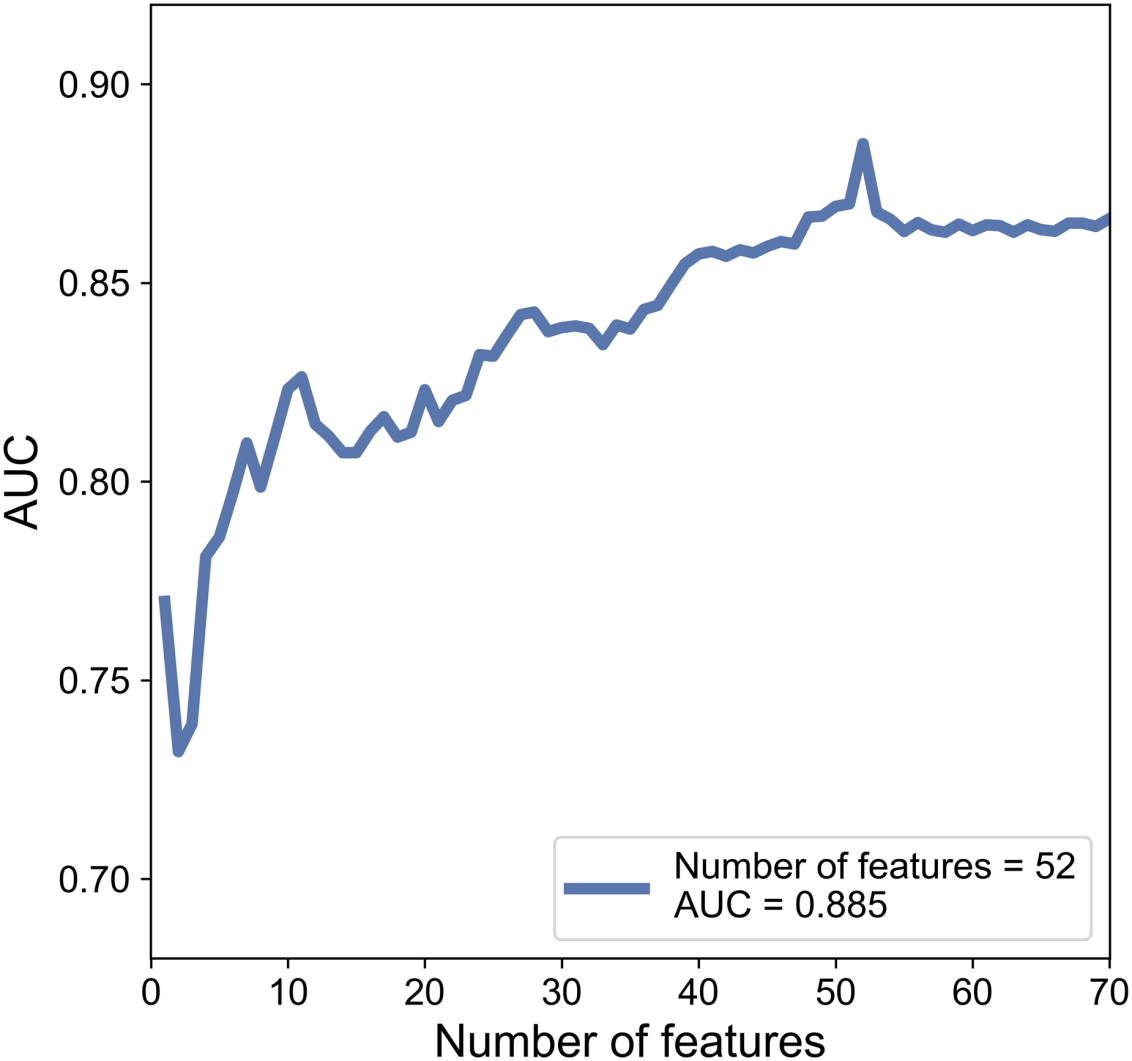
The change of AUC with the number of features.

While training, each cranial US ROI was extracted 350 features based on grayscale, Wavelet_A, Wavelet_V, Wavelet_D, and Wavelet_H modes. Then 350 features were ranked by the index of SRC. Finally, SVM based C-SVC was introduced to predict WMI according to a different number of features. After the examination, we find that while using 52 features, the model performed the best with an AUC of 0.885. The change of AUC with the number of features is shown in the Figure 5.

**Figure 6:**
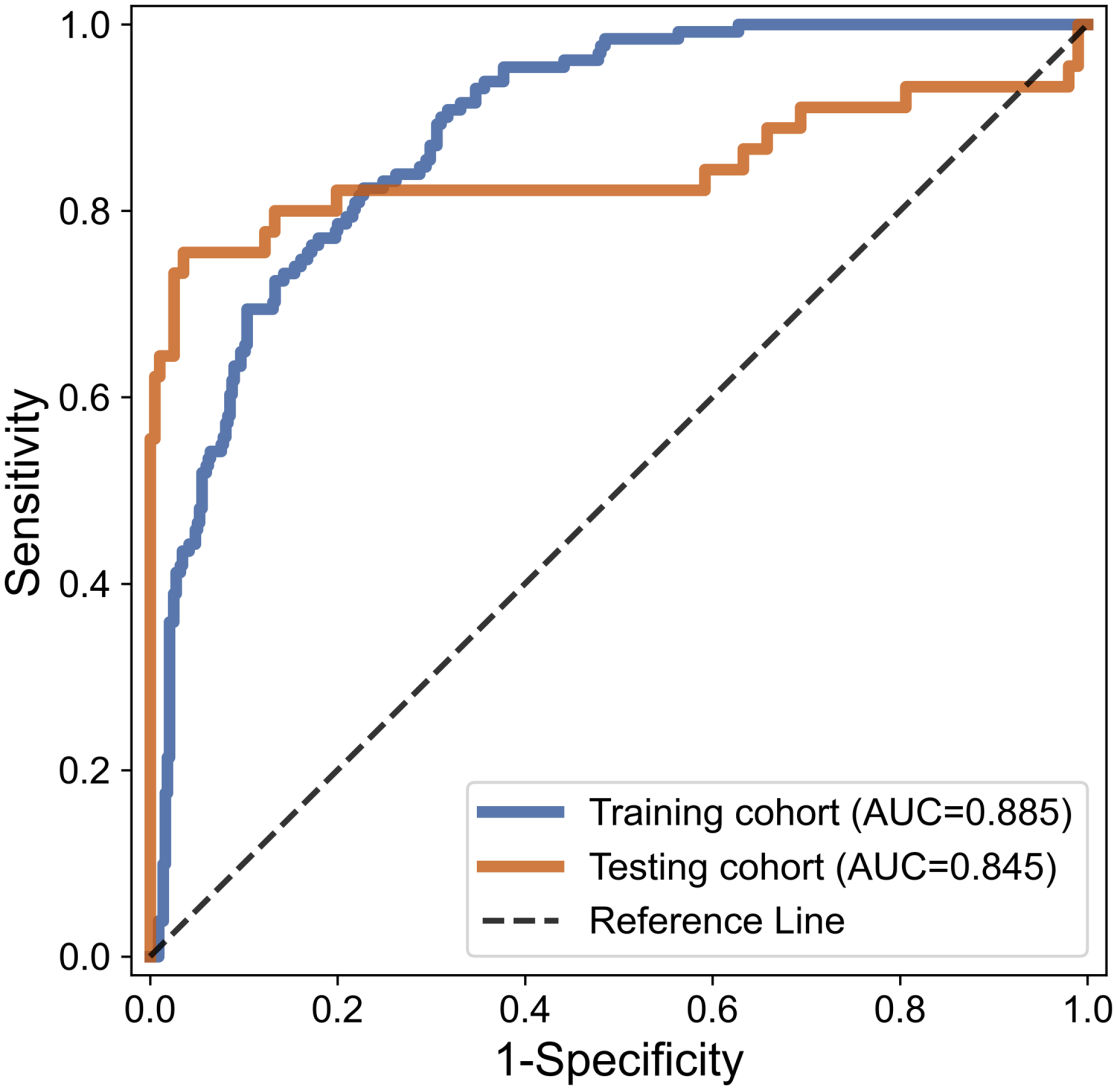
ROC curves of SVM based C-SVC for WMI status prediction in the training and testing set.

While testing, consistent with the training section, 350 features were extracted from cranial ultrasound ROIs based on 5 wavelet transformed modes and were ranked by the index of SRC. We used the top 52 features in SVM classifier. As a result, the AUC of SVM based C-SVC reached 0.845 in the testing cohort. The result is listed in Table 2. Figure 6 illustrated the ROC curves of SVM based C-SVC in the training and testing cohort.

### 3.2 White matter segmentation based on SDL-Net

In clinical trials, manual segmentation of ultrasound images by radiologists will consume a lot of time. In this part, in order to reduce the labor cost of radiologists and avoid labeling manually, we tried to use SDL-Net to implement the white matter segmentations autonomous by computer.

**Figure 7:**
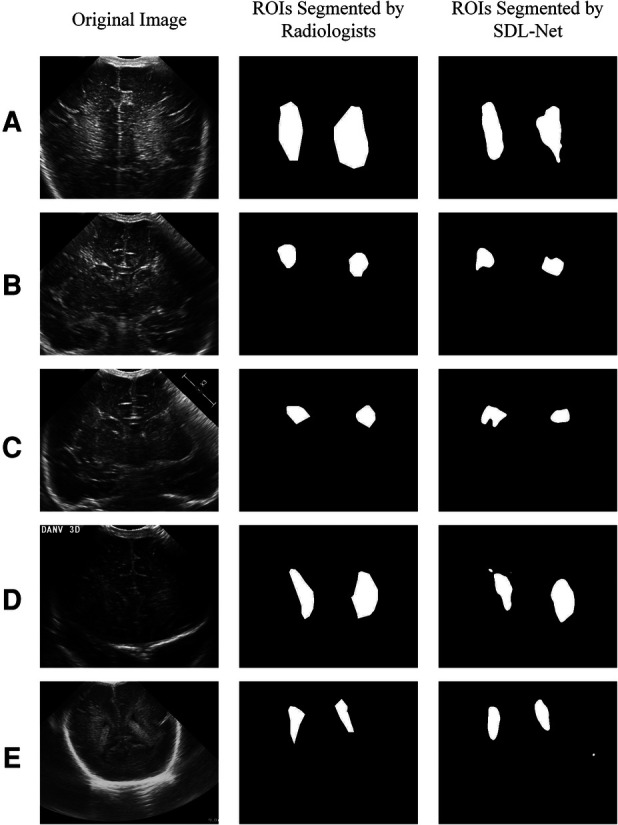
White matter ROIs segmented by SDL-Net.

We used training cohort to train the parameters in SDL-Net, and then used testing cohort to evaluate the segmented results. As shown in Figure 7, we can see that the segmented result by SDL-Net is slightly different from the golden standard ROIs segmented by radiologists. The result is listed in Table 4. It can be observed that our method on testing cohort has an overall Dice of 0.73, an accuracy of 0.80, and an IoU of 0.72.

**Table 2:** The corresponding quantitative indexes for WMI status prediction in the training and testing cohort.

Dataset	AUC	ACC	SENS	SPEC	LR+	LR−
Training cohort	0.885	78.27	82.44	77.24	3.62	0.23
Testing cohort	0.845	87.88	75.56	96.43	21.16	0.25

**Table 3:** The corresponding quantitative indexes for WMI status prediction based on different ROIs in the testing cohort.

ROI used	AUC	ACC	SENS	SPEC	LR+	LR−
ROIs segmented by radiologists	0.845	87.88	75.56	96.43	21.16	0.25
ROIs segmented by SDL-Net	0.819	84.85	64.44	95.92	15.79	0.37

**Figure 8:**
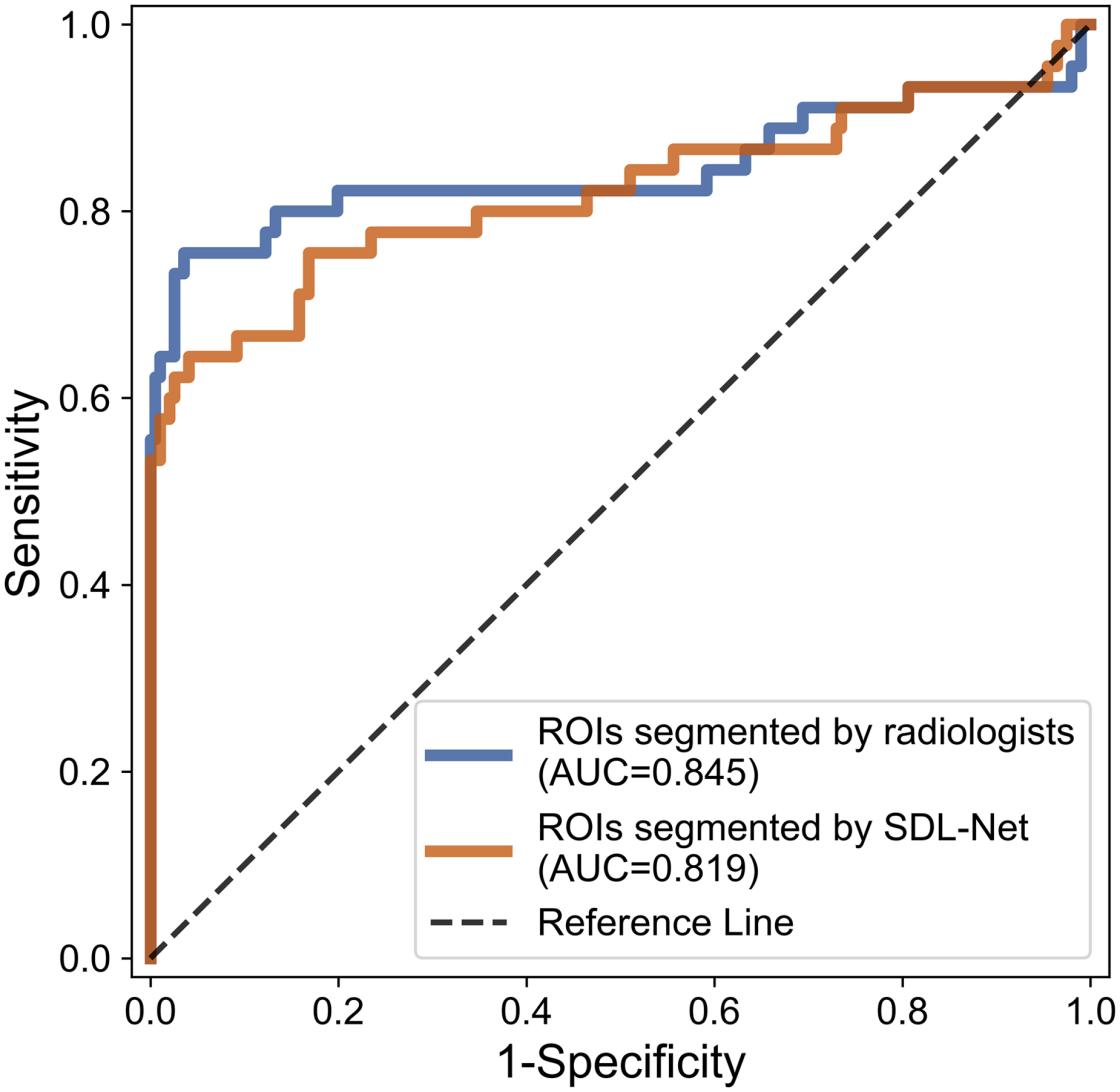
ROC curves for WMI status prediction based on different ROIs in the testing cohort.

Then we used our segmented result in ultrasound radiomics diagnostic system. The result is listed in Table 3. As shown, AUC of SVM based C-SVC reached 0.819 in the testing cohort. The ROC curves of SVM based C-SVC in testing cohort is shown in Figure 8.

### 3.3. Multi-task learning based on MTDL-Net

Multi-task learning (MTL) spontaneously learns multiple related tasks, utilizing both generic information shared across tasks and information specific to each task (30). In this part, MTDL-Net achieved automatic segmentation of infant cranial ultrasound images and used the segmented information to achieve prediction of WMI.

**Figure 9:**
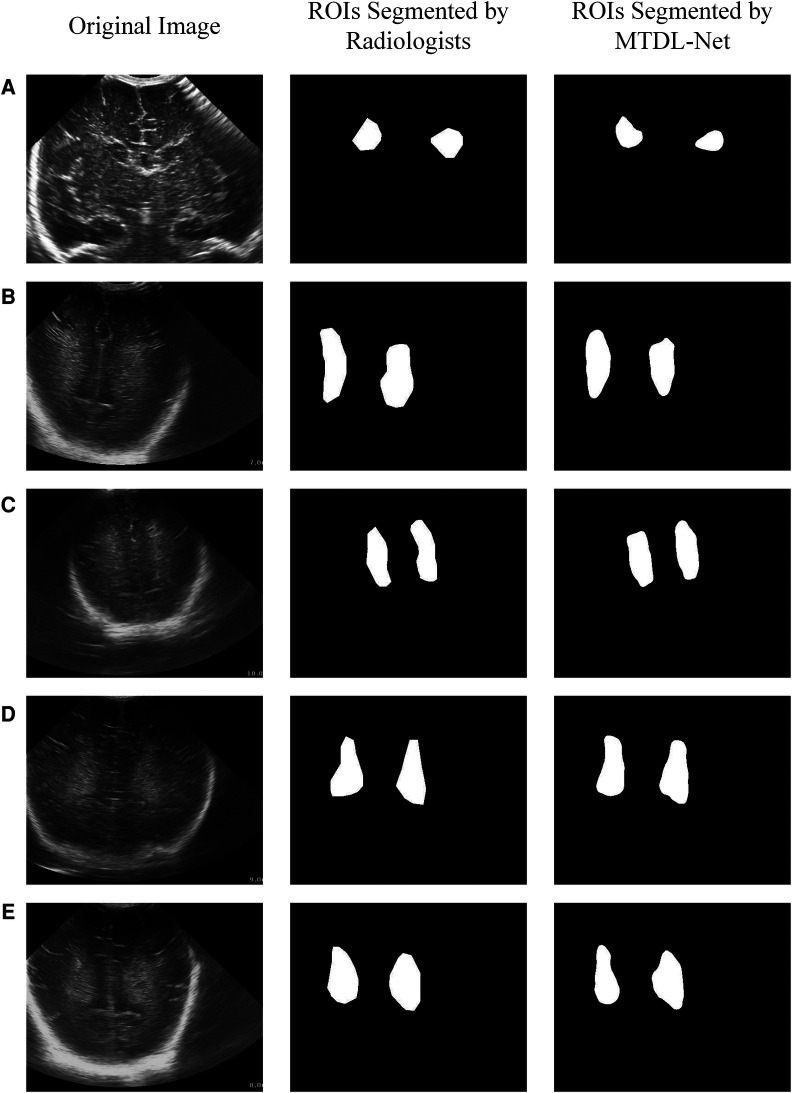
White matter ROIs segmented by MTDL-Net.

We used training cohort to train the parameters in MTDL-Net, and then used testing cohort to evaluate the segmented results and prediction results. Part of the segmented results shows in Figure 9. As shown, the segmented result by MTDL-Net achieved better performance than SDL-Net. Table 4 shows the results of our methods. It can be observed that segmented result on testing cohort has an overall Dice of 0.78, an accuracy of 0.81, and an IoU of 0.82.

**Table 4:** The corresponding quantitative indexes for white matter segmentation based on different models in the testing cohort.

Model	DICE	ACC	IoU
SDL-Net	0.73	0.80	0.72
MTDL-Net	0.78	0.81	0.82

**Figure 10:**
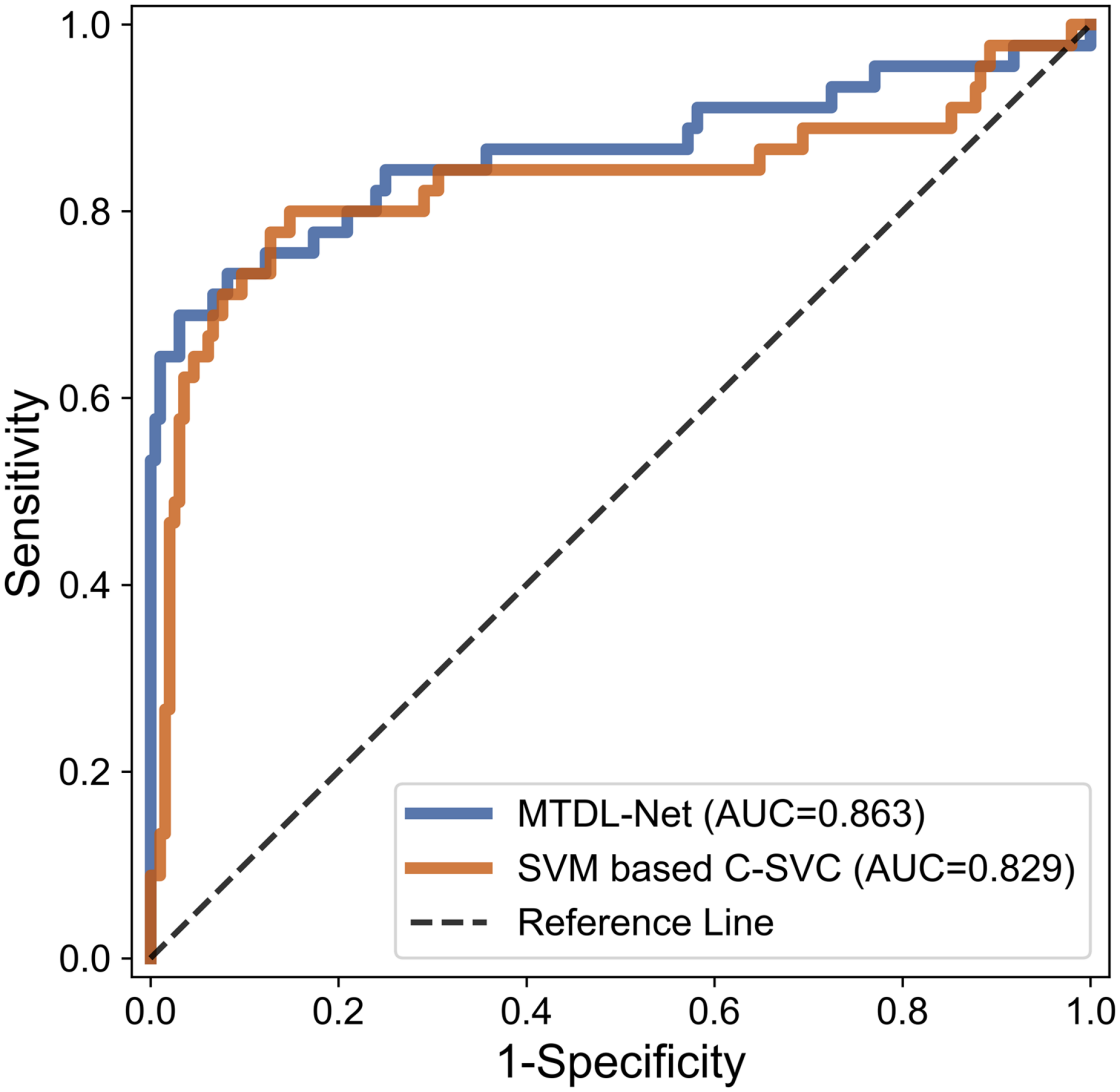
ROC curves for WMI status prediction based on different classifier in the testing cohort.

In the WMI prediction task, we sent segmented result by MTDL-Net into ultrasound radiomics diagnostic system to predict WMI as a comparison. The result is listed in Table 5. As shown, AUC of our method reached 0.863 in the testing cohort, while AUC of SVM based C-SVC was 0.829. The ROC curves of our method and SVM based C-SVC in testing cohort is shown in Figure 10.

**Table 5:** The corresponding quantitative indexes for WMI status prediction based on different classifiers in the testing cohort.

Classifier	AUC	ACC	SENS	SPEC	LR+	LR−
SVM based C-SVC	0.829	80.30	80.00	85.20	5.41	0.23
MTDL-Net	0.863	87.88	68.89	96.94	22.50	0.32

Meanwhile, in order to evaluate the MTDL-Net for multi-task learning, we also implemented the 5-folds cross validation in our data-driven diagnostic strategy. The details of this experiment are described in Supplementary Material. The average DICE coefficient of white matter segmentation in 5-folds cross validation is 0.76. The average AUC of WMI status prediction in 5-folds cross validation is 0.843. 5-fold cross-validation reduced the variance in the estimate of model performance. The result of 5-fold cross-validation demonstrated that MTDL-Net for multi-task learning showed great promising both in white matter segmentation and WMI status prediction in our data-driven diagnostic strategy.

## 4. Discussion

White matter injury is a common brain injury disorder in preterm infants, with severe cases it will lead to periventricular leukomalacia. At present, there is no definite and effective treatment for this disease, and the clinical goal is mainly focused on early diagnosis and intervention. Imaging is the only diagnostic tool for cerebral white matter injury.

The development of cranial ultrasound technology in newborns has opened up a new way for clinicians to understand intracranial lesions *in vivo*. Non-invasive, painless, inexpensive, easy-to-perform, and intuitive, cranial ultrasound testing technology has so far become an indispensable routine method for the diagnosis of intracranial diseases in high-risk infants, and is widely used in the examination process of infant cerebral WMI. The need for a non-destructive and efficient method to predict WMI in infants through ultrasound images is urgent because the area of WMI is too blurred and poorly characterized.

**Figure 11:**
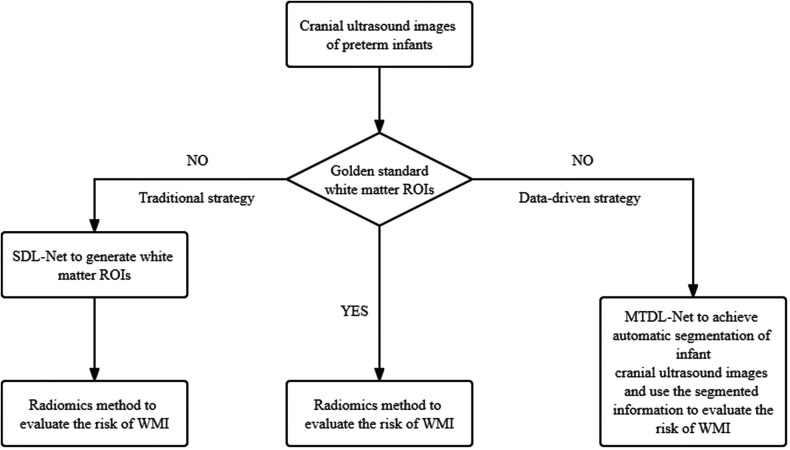
Overview of our data-driven diagnostic strategy.

The major novelty of this paper is that we established a data-driven diagnostic strategy to evaluate the risk of WMI based on cranial ultrasound images. In short, if we already have golden standard white matter ROIs, we can only use radiomics method to evaluate the risk of WMI. But if we don't have golden standard white matter ROIs, we can use MTDL-Net to achieve automatic segmentation of infant cranial ultrasound images and use the segmented information to evaluate the risk of WMI simultaneously. The overview of our data-driven diagnostic strategy is presented in Figure 11. More details are described in Supplementary Material.

In order to implement our data-driven diagnostic strategy, this study attempted a set of methods for preterm infants WMI prediction, starting from the traditional ultrasound radiomics diagnostic system to predict WMI, and finally investigating deep learning methods for automatic segmentation and multi-task learning. The main findings as follows:

### 4.1. WMI prediction

This study implemented the prediction of WMI in preterm infants through B-mode ultrasound images based on an ultrasound radiomics diagnostic system. To utilize the information contained in B-mode ultrasound images, we attempted a high-throughput feature description system for infant cranial ultrasound images, extracting a total of 350 high-throughput features including histogram, GLCM, GLRLM, GLSZM and NGTDM from the original grayscale ultrasound image and four wavelet transformed modes respectively. The feature selection is then performed using SRC combined with 100 times bootstrap technique (31). Finally, the classification model was built using a SVM based C-SVC classifier.

The ultrasound radiomics diagnostic system built with 52 features after feature selection performed the best, with an AUC of 0.845, ACC of 0.8788, SENS of 0.7556, and SPEC of 0.9643 in the testing cohort. The experiments demonstrated that ultrasound radiomics diagnostic system for cranial WMI in preterm infants is effective and has sufficient clinical application value.

### 4.2. White matter segmentation

In clinical trials, manual segmentation of ultrasound images by radiologists will consume a lot of time. And different radiologists with different experience may produce quite different ROIs in the same ultrasound images (32). There is an urgent need for a consistent, nondestructive and efficient method for white matter segmentation in preterm infants and guiding the clinical diagnosis and treatment process.

U-Net was proved of great performance in many clinical scenarios such as segmentation of brain vessel status(33). TransUNet and Swin-Unet are Transformer-based network, which need much more training data rather than CNN-based network. Comparing to other state-of-the-art methods, U-Net adopted in this paper obtains the best segmentation performance under the premise of amount of our data. Models based on the Transformer framework may have better performance after expanding the sample size. Therefore, U-Net based SDL-Net was applied to achieve automatic segmentation of infant cranial brain white matter ROIs based on ultrasound images. Dice coefficient of white matter segmentation reached 0.73 and the accuracy reached 0.80 in the testing set.

In order to evaluate the performance of the segmented ROIs by SDL-Net, we utilized the segmented results as the input of ultrasound radiomics diagnostic system to predict the WMI of preterm infants. As we can see, the AUC of the classifier reached 0.819 in the testing cohort, which is only slightly lower than the experiment with the golden standard ROIs segmented by radiologists.

### 4.3. Multi-task learning

Most deep learning methods use useful information from historical data to help analyze future data and typically requires large amounts of labeled data for training. However, certain application (e.g., medical image analysis) cannot meet this requirement because it requires a lot of manual labor to label data. In these cases, multi-task learning is a good approach that uses useful information from other related tasks to alleviate data sparsity problem (34).

Based on the assumption that all tasks or at least some of them are relevant (30), MTL aims to share useful information contained in multiple tasks to build more accurate models which work better empirically and theoretically than learning each tasks independently.

In this part, MTDL-Net is applied to achieve automatic segmentation of infant cranial US images and used the segmented information to achieve prediction of WMI. MTDL-Net extracted the feature map from the whole image through FPN and ResNet50, selected the candidate regions through RPN, obtained the corresponding feature map of each candidate regionthrough ROI Align. Finally, the segmentation mask was output through FCN, and B-Box and classification labels were output through the fully connected layer.

In the segmentation task, MTDL-Net performed best with Dice coefficient of 0.78, which is better than SDL-Net. In the classification task, MTDL-Net performed best with AUC of 0.863, also better than the ultrasound radiomics diagnostic system. And within the ROIs segmented by MTDL-Net, ultrasound radiomics diagnostic system had equivalent performance comparing with the input of golden standard ROIs segmented by radiologists. Therefore, MTDL-Net can simultaneously achieve the ROI segmentation of white matter based on ultrasound images and prediction of WMI in preterm infants.

There were also some limitations in our study. First, in practical clinical applications, ultrasound examinations are in the form of video scanning plus preservation of static images. Therefore, if the ultrasound video data can be processed directly instead of using static images, the automaticity of the system will be improved and enhanced (35, 36). In addition, due to the limited number of WMI cases, preterm infants with PVL were not categorized together for comparison. Further studies are needed to determine the potential differences in the predictive efficacy of WMI and PVL.

In conclusion, this paper presents a diagnostic system for white matter injury in preterm infants. The system combines multi-task deep learning and traditional radiomics features to achieve automatic detection of white matter regions on the one hand, and design a fusion strategy of deep learning features and manual radiomics features on the other hand to obtain stable and efficient diagnostic performance.

## Data Availability

The data that support the findings of this study are available from the corresponding author on reasonable request.
