# Supplementary material for: White matter injury detection based on preterm infant cranial ultrasound images
Source: Front Pediatr. 2023 Apr 20;11:1144952. doi: 10.3389/fped.2023.1144952 (PMC10157025; doi:10.3389/fped.2023.1144952)
Supplement: Supplementary file 1 [file Datasheet1.pdf]

Supplementary Material

White Matter Injury Detection Based on Preterm Infant Cranial Ultrasound Images

Juncheng Zhu†, Shifa Yao†, Zhao Yao, Jinhua Yu, Zhaoxia Qian*, and Ping Chen*

† These authors contributed equally to this study and shared first authorship;

* These authors contributed equally to this study and shared corresponding authorship.

*** Correspondence:**
Zhaoxia Qian, zhaoxiaqian@163.com;
Ping Chen, 2429859650@qq.com

1 Evaluation Index

The AUC was used as an index for the evaluation of the diagnostic performance. The sensitivity (SENS), specificity (SPEC), accuracy (ACC), and positive and negative diagnostic likelihood ratios (LR+, LR−) were calculated. We adopted Dice coefficient (Dice) and Intersection over Union (IoU) to evaluate the performance of segmentation. The definitions of these indexes are shown below.

$$Accuracy\ (ACC) = \frac{TP + TN}{TP + FP + TN + FN}$$

$$Sensitivity\ (SENS) = \frac{TP}{TP + FN}$$

$$Specificity\ (SEPC) = \frac{TN}{FP + TN}$$

$$Positive\ predictive\ value\ (PPV) = \frac{TP}{TP + FP}$$

$$Negative\ predictive\ value\ (NPV) = \frac{TN}{TN + FN}$$

$$Positive\ diagnostic\ likelihood\ ratios\ (LR+) = \frac{SENS}{1 - SEPC}$$

$$Negative\ diagnostic\ likelihood\ ratios\ (LR-) = \frac{1 - SENS}{SEPC}$$

$$Dice\ coefficient\ (Dice) = \frac{2 \times area\ of\ overlapped}{total\ area}$$

$$Intersection\ over\ Union\ (IoU) = \frac{Intersection\ area}{Union\ area} = \frac{TP}{TP + FP + FN}$$

where TP stands for the number of positive patients who were got right classification, FP stands for the number of positive patients who were got wrong classification, TN stands for the number of negative patients who were got right classification, FN stands for the number of negative patients who were got wrong classification.

AUC represents the area under the receiver operating characteristic curve (ROC). The horizontal and vertical coordinates of ROC are 1-SEPC and SENS respectively. The closer the ROC curve is to the top left corner, the more accurate the model is.

2 Data-driven Diagnostic Strategy

The he novelty of this paper is the innovation of model building process according to the data situation rather than the innovation of deep learning networks, so the major novelty of this paper is that we established a data-driven diagnostic strategy to evaluate the risk of WMI based on cranial ultrasound images. In short, if we already have golden standard white matter ROIs, we can only use radiomics method to evaluate the risk of WMI. But if we don't have golden standard white matter ROIs, we can use MTDL-Net to achieve automatic segmentation of infant cranial ultrasound images and use the segmented information to evaluate the risk of WMI simultaneously.

As a comparison, we also implemented the traditional strategy which is firstly to use SDL-Net to generate white matter ROIs, and then use radiomics method to evaluate the risk of WMI. The results show that our data-driven diagnostic strategy outperformed than traditional strategy, which reached state-of-the-art in the risk of WMI evaluation task. The overview of our data-driven diagnostic strategy is presented in Supplementary Figure 1.

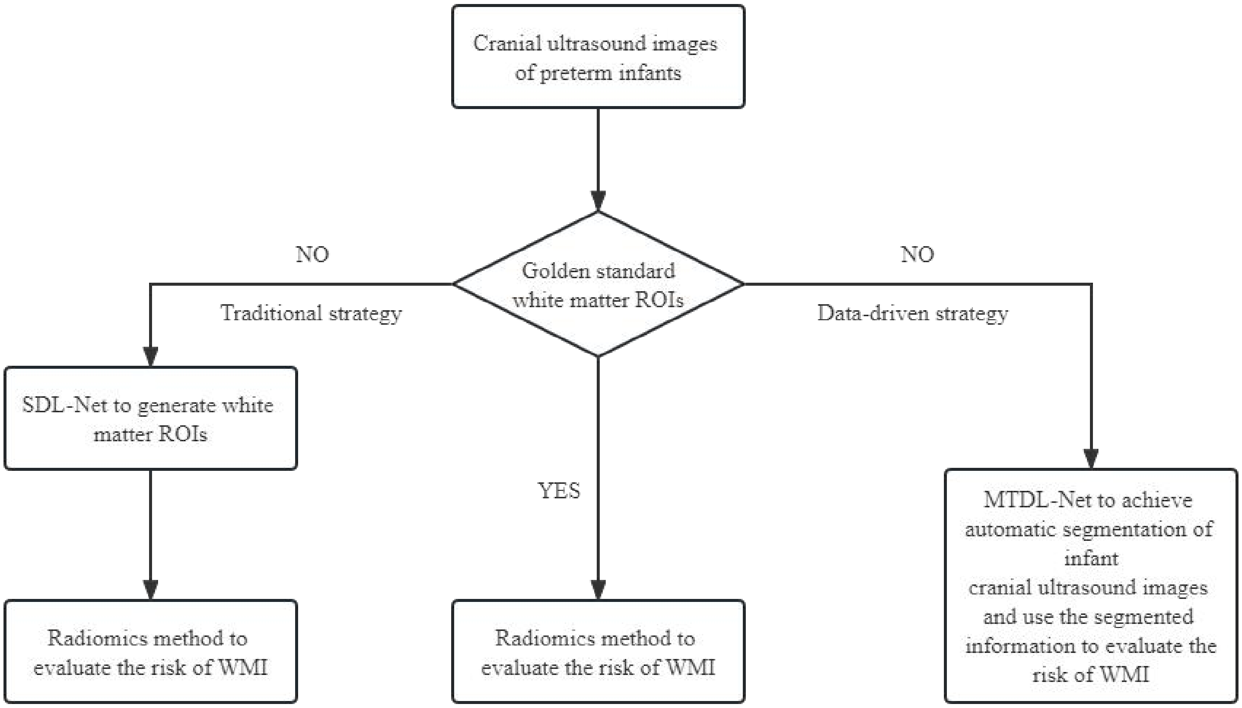

Supplementary Figure 1. Overview of our data-driven diagnostic strategy.

First of all, we proposed a method based on ultrasound radiomics to predict the WMI risk. In radiomics method, we need to input white matter region of interest (ROIs) of preterm infants' cranial ultrasound images. These ROIs were golden standard ROIs which were segmented manually by radiologists.

However, in clinical trials, manual segmentation of ultrasound images by radiologists will consume a lot of time. In order to reduce the labor cost of radiologists and avoid labeling manually, we proposed SDL-Net to perform the white matter segmentations autonomous. Therefore, SDL-Net is used to generate white matter ROIs to reduce the labor cost of radiologists in clinical trials.

Finally, based on that segmentation of white matter and evaluation the risk of WMI are related tasks, which is inherently suitable for multi-task learning, we proposed MTDL-Net to achieve automatic segmentation of infant cranial ultrasound images and to use the segmented information to achieve prediction of WMI.

In conclusion, our data-driven diagnostic strategy maximizes the use of existing data that have been preprocessed by professionals. In addition, it also solved the modeling problem without radiologists' annotation. Our data-driven diagnostic strategy improved WMI diagnosis performance by 4.4% in AUC and 3.1% in ACC compared to the traditional strategy with best available deep learning method.

3 Data augmentation techniques

In our manuscript, we have already used augmentation techniques to balance and augment the training dataset. Supplementary Figure 2 shows the details of our data augmentation techniques.

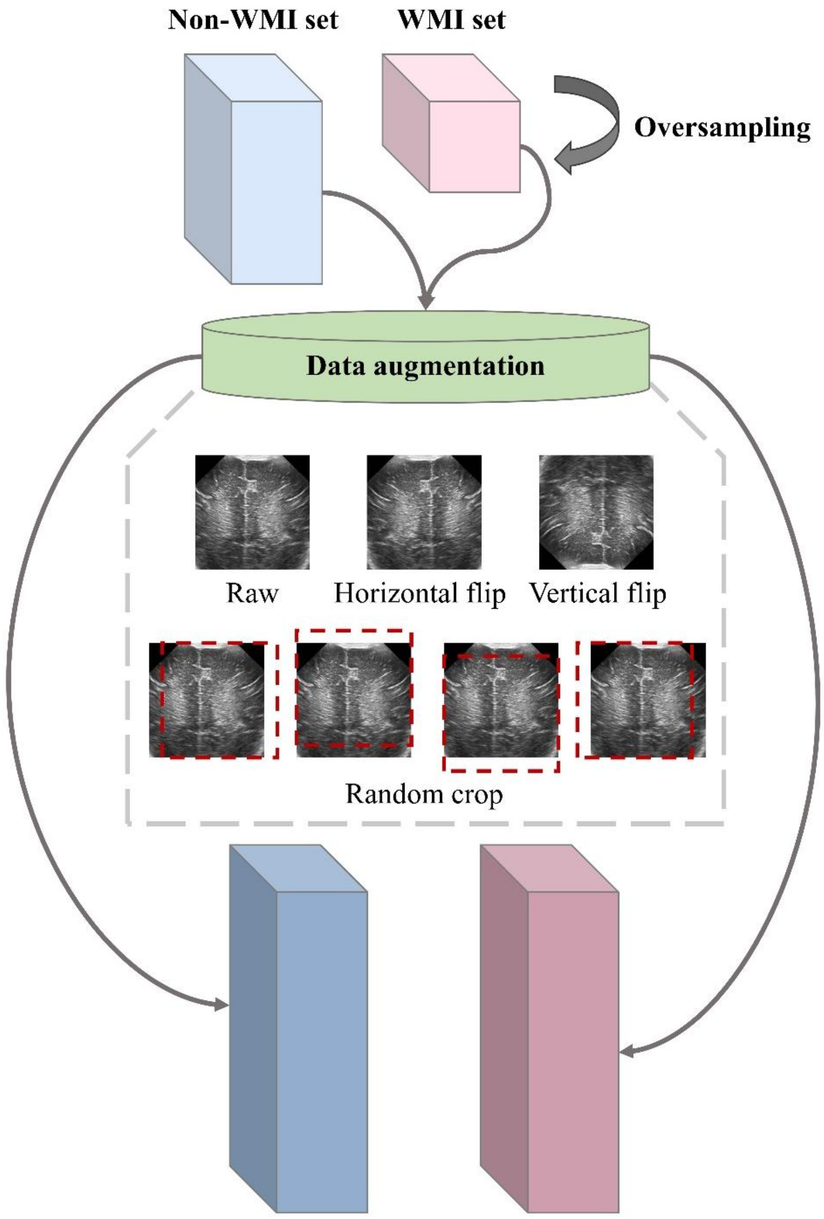

Supplementary Figure 2. Overview of our data augmentation techniques.

The study population consisted of 158 preterm infants with 807 cranial US images. 126 cases in the data set were normal preterm infants and 32 cases were preterm infants with WMI. We randomly split the data set into training cohort (110 preterm infants with 566 cranial US images) and testing cohort (48 preterm infants with 241 cranial US images) at a ratio of 7:3. The training cohort consists of 88 normal preterm infants (435 cranial US images) and 22 preterm infants with WMI (131 cranial US images). The testing cohort consists of 38 normal preterm infants (196 cranial US images) and 10 preterm infants with WMI (45 cranial US images).

While training, we implemented some tricks to optimize the unbalanced dataset and augment the training dataset. First of all, in order to improve the generalization ability considering the limited medical data, we applied oversampling strategy to augment the data which belong to preterm infants with WMI during training. Additionally, the training set was artificially augmented by random translation, rotation and flipping from the original images to prevent overfitting of the networks due to the limited training dataset. Finally, in MTDL-Net, we used focal loss[1] to prevent misclassification bias due to the unbalanced dataset. The definition of focal loss is shown below.

$$FL(p) = \begin{cases} -\alpha(1-p)^{\gamma}\log(p),\ y=1 \\ -(1-\alpha)p^{\gamma}\log(1-p),\ y=0 \end{cases}$$

Where, p is the prediction probability, α is the weight of the positive sample, and $(1-p)^{\gamma}$ is the modulation coefficient of the difficult sample in the positive sample. When p approaches 0, the modulation coefficient approaches 1, contributing more to the total loss. Therefore, the model's attention to difficult samples can be controlled by γ. Increasing the modulation coefficient γ reduces the of easy samples' weight value, making the training process pay more attention to difficult samples.

4 Segmentation Deep Learning Model

For segmentation tasks, Transformer has the advantage of better non-local analysis than CNN-based methods. Therefore, we increased the comparison of TransUNet[2] and Swin-Unet[3], which are both outstanding methods in segmentation recently. In order to find the most suitable segmentation deep learning network (SDL-Net), we compared FCN[4], U-Net[5], TransUNet[2] and Swin-Unet[3] for white matter segmentations. The results are listed in the Supplementary Table 1. The results of the method we used are bolded.

Supplementary Table 1. The corresponding quantitative indexes for white matter segmentation based on different models in the testing cohort.

Model	DICE	ACC	IoU
FCN [2015]	0.66	0.70	0.55
**U-Net [2015]**	**0.73**	**0.80**	**0.72**
TransUNet [2021]	0.70	0.76	0.65
Swin-Unet [2021]	0.69	0.78	0.63

As shown, U-Net performed best with the DICE coefficient of 0.73. TransUNet and Swin-Unet are Transformer-based network, which need much more training data rather than CNN-based network. Meanwhile, U-Net which is modified from FCN is proved great performance in medical image analysis. After comparison, U-Net adopted in this paper obtains the best segmentation performance under the premise of amount of our data. Models based on the Transformer framework may have better performance after expanding the sample size. As a result, we chose U-Net as our segmentation deep learning network in this paper.

5 Segmentation Deep Learning Model

In order to find the most suitable multi-task deep learning network (MTDL-Net), we compared Fast R-CNN[6], Faster R-CNN[7], Mask R-CNN[8] and RS-Net[9] for white matter segmentations and WMI status prediction. Based on the previous experiments, CNN based network is more suitable in our case. So we did not choose transformer based network as a comparison. Instead, we chose RS-Net[9] which showed great performance in segmentation and prediction of Glioma tasks as a comparison. The segmentation results are listed in the Supplementary Table 2 and prediction results are listed in Supplementary Table 3. The results of the method we used are bolded.

Supplementary Table 2. The corresponding quantitative indexes for white matter segmentation based on different multi-task deep learning networks in the testing cohort.

Model	DICE	ACC	IoU
Fast R-CNN [2015]	0.68	0.74	0.66
Faster R-CNN [2015]	0.69	0.77	0.69
**Mask R-CNN [2020]**	**0.73**	**0.79**	**0.74**
RS-Net [2022]	0.78	0.81	0.82

Supplementary Table 3. The corresponding quantitative indexes for WMI status prediction based on different multi-task deep learning networks in the testing cohort.

Model	AUC	ACC	SENS	SPEC	LR+	LR-
Fast R-CNN [2015]	0.806	0.80	66.67	85.71	4.67	0.39
Faster R-CNN [2015]	0.819	0.82	77.78	77.55	3.46	0.29
**Mask R-CNN [2020]**	**0.838**	**80.30**	**66.67**	**91.84**	**8.17**	**0.36**
RS-Net [2022]	0.863	87.88	68.89	96.94	22.50	0.32

The ROC curves of WMI status prediction based on different multi-task deep learning networks in testing cohort is shown in Supplementary Figure 3. As shown, Mask R-CNN performed best both in white matter segmentation and WMI status prediction tasks. As a result, we chose Mask R-CNN as our multi-task deep learning network in this paper.

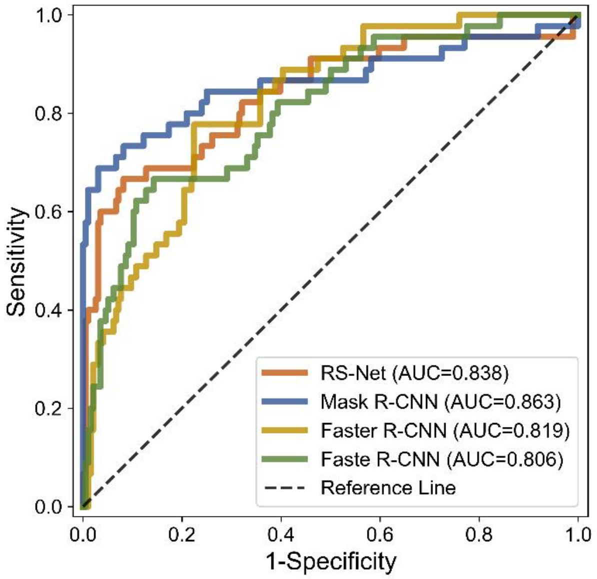

Supplementary Figure 3. The ROC curves of WMI status prediction based on different multi-task deep learning networks in testing cohort.

6 5-fold cross validation

We implemented the 5-fold cross validation to evaluate our proposed method. The study population consisted of 158 preterm infants with 807 cranial US images. 126 cases in the data set were normal preterm infants and 32 cases were preterm infants with WMI. In order to ensure each fold contains about the same amount of data of each type, we randomly split the normal preterm infants and preterm infants with WMI into 5 folds, respectively. The baseline characteristics of the patients are summarized in Table R1.

Supplementary Table 4. The baseline characteristics of the patients.

Folds	Patients	Non-WMI	WMI
Fold 1	32 (160)	26 (133)	6 (27)
Fold 2	32 (141)	25 (105)	7 (36)
Fold 3	32 (166)	25 (114)	7 (52)
Fold 4	31 (178)	25 (149)	6 (29)
Fold 5	31 (162)	25 (130)	6 (32)
**Sum**	**158 (807)**	**126 (631)**	**32 (176)**

**The number of cranial US images are listed in parentheses.*

While training, we also implemented an asymmetric cyclic sampling strategy to optimize the unbalanced dataset and augment the dataset. And we also artificially augmented the dataset by random translation, rotation and flipping from the original images to prevent overfitting of the networks due to the limited dataset.

We implemented the 5-folds cross validation in our data-driven diagnostic strategy to evaluate the MTDL-Net for multi-task learning. The segmentation results of 5-folds cross validation are listed in Table R2 and the prediction results of 5-folds cross validation are listed in Table R3.

Supplementary Table 5. The corresponding quantitative indexes for white matter segmentation of 5-folds cross validation.

Folds	DICE	ACC	IoU
Fold 1	0.75	0.77	0.76
Fold 2	0.80	0.82	0.79
Fold 3	0.78	0.79	0.74
Fold 4	0.71	0.74	0.72
Fold 5	0.74	0.78	0.75
**Average**	**0.76**	**0.78**	**0.75**

** Each row means that fold as testing set.*

Supplementary Table 6. The corresponding quantitative indexes for WMI status prediction of 5-folds cross validation.

Folds	AUC	ACC	SENS	SPEC	LR+	LR-
Fold 1	0.854	89.38	70.37	93.98	11.70	0.32
Fold 2	0.845	92.20	75.00	98.10	39.37	0.25
Fold 3	0.826	84.94	71.15	91.23	8.11	0.32
Fold 4	0.841	84.27	72.41	86.58	5.39	0.32
Fold 5	0.850	88.27	78.12	90.77	8.46	0.24
**Average**	**0.843**	**87.81**	**73.41**	**92.13**	**14.61**	**0.29**

** Each row means that fold as testing set.*

As shown, MTDL-Net performed great both in white matter segmentation and WMI status prediction. The average DICE coefficient of white matter segmentation in 5-folds cross validation is 0.76. The average AUC of WMI status prediction in 5-folds cross validation is 0.843. 5-fold cross-validation reduces the variance in the estimate of model performance. The result of 5-fold cross-validation demonstrated that MTDL-Net for multi-task learning shows great promising both in white matter segmentation and WMI status prediction in our data-driven diagnostic strategy. The ROC curves of WMI status prediction of 5-folds cross validation are shown in Figure R1.

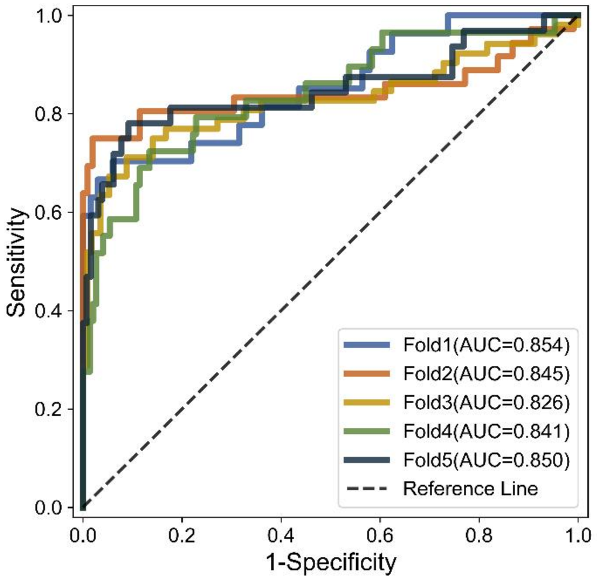

Supplementary Figure 4. The ROC curves of WMI status prediction of 5-folds cross validation.

Reference

1. Lin, T.Y., et al., *Focal Loss for Dense Object Detection.* Ieee Transactions on Pattern Analysis and Machine Intelligence, 2020. **42**(2): p. 318-327.
2. Long, J., E. Shelhamer, and T. Darrell, *Fully Convolutional Networks for Semantic Segmentation.* 2015 Ieee Conference on Computer Vision and Pattern Recognition (Cvpr), 2015: p. 3431-3440.
3. Ronneberger, O., P. Fischer, and T. Brox, *U-Net: Convolutional Networks for Biomedical Image Segmentation.* Medical Image Computing and Computer-Assisted Intervention, Pt Iii, 2015. **9351**: p. 234-241.
4. Chen, J., et al., *TransUNet: Transformers Make Strong Encoders for Medical Image Segmentation.* 2021.
5. Cao, H., et al., *Swin-Unet: Unet-like Pure Transformer for Medical Image Segmentation.* 2021.
6. Girshick, R., *Fast R-CNN.* 2015 Ieee International Conference on Computer Vision (Iccv), 2015: p. 1440-1448.
7. Ren, S.Q., et al., *Faster R-CNN: Towards Real-Time Object Detection with Region Proposal Networks.* Advances in Neural Information Processing Systems 28 (Nips 2015), 2015. **28**.
8. He, K.M., et al., *Mask R-CNN.* Ieee Transactions on Pattern Analysis and Machine Intelligence, 2020. **42**(2): p. 386-397.
9. van der Voort, S.R., et al., *Combined molecular subtyping, grading, and segmentation of glioma using multi-task deep learning.* Neuro-Oncology, 2022.
